# Racial/Ethnic Differences in Loneliness Among Older Adults: The Role of Income and Education as Mediators

**DOI:** 10.1093/geroni/igae068

**Published:** 2024-07-19

**Authors:** Harry Owen Taylor, Yu-Chih Chen, Kazumi Tsuchiya, Thomas K M Cudjoe, Weidi Qin, Ann W Nguyen, Arka Roy

**Affiliations:** Factor-Inwentash Faculty of Social Work, University of Toronto, Toronto, Ontario, Canada; Department of Social Work and Social Administration, The University of Hong Kong, Pokfulam, Hong Kong SAR, China; Dalla Lana School of Public Health, University of Toronto, Toronto, Ontario, Canada; Division of Geriatric Medicine and Gerontology, Johns Hopkins University School of Medicine, Baltimore, Maryland, USA; Sandra Rosenbaum School of Social Work, University of Wisconsin-Madison, Madison, Wisconsin, USA; Jack, Joseph and Morton Mandel School of Applied Social Sciences, Case Western Reserve University, Cleveland, Ohio, USA; Dalla Lana School of Public Health, University of Toronto, Toronto, Ontario, Canada

**Keywords:** Ethnicity, Race, Socioeconomic status, Wealth

## Abstract

**Background and Objectives:**

Loneliness is a major public health concern; however, limited research has examined the mechanisms contributing to racial/ethnic inequities in loneliness. Race/ethnicity has been hypothesized to be a distal factor influencing loneliness, and racial/ethnic inequities in loneliness may be attributable to socioeconomic factors (e.g., income and education). Our study seeks to confirm these hypotheses by examining mechanisms that contribute to racial/ethnic inequities in loneliness. In other words, if racial/ethnic differences in loneliness among older adults are mediated by income and education.

**Research Design and Methods:**

Data came from the Health and Retirement Study Leave-Behind Questionnaire, 2014–2016. Loneliness was measured by the UCLA 3-item loneliness scale. Race/ethnicity categories were White, Black, and Hispanic/Latino. The mediator variables were household income and education. Multivariable linear regression models were used to determine differences in loneliness by race/ethnicity. The Karlson–Holm–Breen (KHB) mediation method was used to determine if income and education mediated racial/ethnic differences in loneliness.

**Results:**

In models examining income and education together, a complete mediation was found between White and Black older adults, in that income and education completely mediated differences in loneliness between these groups. A partial mediation was found between White and Hispanic, and Black and Hispanic older adults. When examining income and education separately, we found that income solely accounted for racial/ethnic differences in loneliness compared to education.

**Discussion and Implications:**

Our study is the first to explicitly determine if socioeconomic factors mediate race/ethnicity differences in loneliness among a national sample of older adults. These findings illustrate that income may have greater proximate effects for loneliness among older adults in comparison to education. Additionally, these findings can inform evidence-based interventions to reduce loneliness among older adults. Interventions that enhance quality of life and provide opportunities for socialization for racialized low-income older adults may help decrease racial/ethnic inequities in loneliness.

**Translational Significance:** Our study is among the first to empirically examine mechanisms that create and sustain racial/ethnic inequities in loneliness among older adults. Findings from this study can inform future interventions to reduce racial/ethnic inequities in loneliness among older adults. Racial/ethnic inequities in loneliness among older adults may be attributable to income. Given income is a flexible resource that influences multiple domains, interventions that improve social connections, quality of life, and provide greater opportunities for socialization (including peer-to-peer outreach, volunteer, and community navigator interventions) for racialized/ethnic and low-income older adults may help reduce inequities in loneliness.

## Background and Objectives

Loneliness is a distressing psychosocial condition that profoundly influences health and well-being (National Academies of Sciences, Engineering, and Medicine, 2020). It is defined as having low-quality social relationships, where there is a deficit between the quality of an individual’s current social relationships and the relationships they would like to have (Peplau & Perlman, 1979). Loneliness is also a major public health concern. Numerous prominent health and human services organizations, foundations, and institutions, including the World Health Organization (World Health Organization, 2021), the United States’ Surgeon General (U.S. Department of Health and Human Services, 2023), the Robert Wood Johnson Foundation (Ladden, 2019), the National Institute on Aging (National Institute on Aging, 2019), the American Psychological Association (Novotney, 2019), the Agency for Healthcare Research and Quality (Veazie et al., 2019), and the American Public Health Association (Klinenberg, 2016) have mobilized to address the loneliness epidemic.

There are notable gaps in loneliness research among older adults, especially for racial/ethnic inequities in loneliness among older adults. This is because there is very limited understanding of which factors contribute to these inequities in loneliness. Race/ethnicity has been viewed as a distal factor that may influence loneliness. More specifically, racial/ethnic inequities in loneliness may be attributed to racial/ethnic differences in socioeconomic factors (Barjaková et al., 2023; Hawkley et al., 2008). Nevertheless, previous empirical investigations have not explicitly examined whether racial/ethnic inequities are driven by socioeconomic factors. The current study advances the empirical research literature on loneliness by examining if income and education account for racial/ethnic differences in loneliness among older adults. Understanding these mechanisms or pathways is critical knowledge for developing interventions to decrease racial/ethnic inequities in loneliness among older adults.

### Loneliness Background

Loneliness is associated with a myriad of negative health outcomes including earlier mortality, greater chronic health conditions, worse self-rated physical and mental health, depressive symptoms, and cognitive decline (National Academies of Sciences, Engineering, and Medicine, 2020). In fact, loneliness is viewed as a social stressor that also increases cellular aging (Hawkley & Cacioppo, 2007). Given the urgency and magnitude of this public health problem, the Surgeon General of the United States recently issued a report on loneliness to raise public awareness and to increase efforts to mitigate this problem (U.S. Department of Health and Human Services, 2023).

Loneliness is an important area of research among older adults as they may experience greater vulnerabilities to loneliness (Elder & Retrum, 2012), and loneliness may have a stronger effect on health in older ages compared to younger ages (Hawkley & Cacioppo, 2007). Rates of loneliness in the United States among older adults range from 34% to 56% (Malani et al., 2023). Older adults may be at greater risk of loneliness due to the multitude of life transitions during this phase of the life course. This includes becoming a caregiver, the loss of family members and friends, potential relocation, retirement, and greater physical and cognitive frailty (Elder & Retrum, 2012). These transitions may prevent older adults from actively engaging with family members and friends, as they may undergo challenges in fully integrating into society.

### Racial/Ethnic Differences in Loneliness Among Older Adults

Race/ethnicity is an important factor in understanding loneliness among older adults. However, findings are mixed and paint a complicated picture (Barjaková et al., 2023). Some studies find no significant differences in racial/ethnic differences in loneliness among older adults (Compernolle et al., 2021; Greenfield & Russell, 2011; Hawkley et al., 2022; Shovestul et al., 2020; Taylor, 2020), whereas others have found Black older adults are lonelier compared to White and Hispanic/Latinx older adults (Henning-Smith et al., 2019; Miyawaki, 2015; Perissinotto et al., 2012; Taylor & Nguyen, 2020). In studies that examine differences between White and Hispanic/Latinx older adults, most report no differences between these two groups (Compernolle et al., 2021; Miyawaki, 2015; Tibiriçá et al., 2022; Tomaka et al., 2006). Nevertheless, some studies do find ethnic differences between these two groups. For example, a study by Perissinotto and colleagues (2012) found Hispanic/Latinx older adults report greater loneliness, whereas other studies find Hispanic/Latinx older adults are less lonely compared to White older adults (Hawkley & Kocherginsky, 2018; Henning-Smith et al., 2019). Given these mixed findings, it is critical to continue investigating this topic to determine whether there are differences in loneliness by race/ethnicity, and under what conditions/contexts these differences emerge. Furthermore, it is important to move from merely understanding that there are racial/ethnic differences in loneliness among older adults to deepening our understanding of *how* and *why* there are racial/ethnic differences in loneliness among older adults.

Race/ethnicity is a central stratifying feature in the United States of America resultant of structural racism and racialization processes, which establish a racial hierarchy (Adkins-Jackson et al., 2022; Chatters et al., 2021; Williams & Collins, 2001). Structural racism refers to the totality of systems in which White people are preferred and privileged in comparison to non-White people (Bailey et al., 2017). Race is socially constructed and does not reflect biological or cultural variations between groups (Smedley & Smedley, 2005). More specifically, differences in health and social exclusion by race/ethnicity are due to cultural, historical, and geographical mechanisms rooted in racism (Bailey et al., 2021). Black and Hispanic/Latinx older adults are more likely to experience greater concentrated disadvantage and marginalization, which translates into living in neighborhoods with limited resources because of racial residential segregation (Williams & Collins, 2001) and consequently, experience greater relational stressors throughout the life course (Chatters et al., 2021). Resultant of structural racism, Black and Hispanic/Latinx older adults are more likely to live in poverty, have lower formal education attainment, and often have worse physical health outcomes in comparison to White older adults (Bailey et al., 2017; Chatters et al., 2021; Williams & Collins, 2001). Black older adults are also significantly less likely to be married and more likely to be kinless in comparison to White older adults (Verdery & Margolis, 2017; Taylor et al., 2023). Given these mechanisms, race/ethnicity as a proxy of structural racism may also be a key factor associated with loneliness among older adults.

Structural racism may also indirectly influence social network size. Studies have found that African Americans typically report having smaller social networks from greater loss of kin and family networks due to death or incarceration (Chatters et al., 2021; Umberson, 2017; Umberson & Donnelly, 2023). These stressors, as a byproduct of racism and racial stratification processes, may also contribute to greater negative interactions with social network members and potentially fewer close relationships, and hence, greater loneliness in these populations (Lee & Bierman, 2019; Negi, 2013; Nguyen et al., 2024; Sutin et al., 2015). The totality of these lived experiences may directly contribute to greater loneliness among racially minoritized populations. Furthermore, the risks factors directly associated with loneliness are often greater among racially minoritized populations, including lower income and lower formal education, lowered likelihood of being married, and more likely to exhibit worse physical health outcomes and well-being (Chatters et al., 2021; Pinquart & Sorensen, 2001; Theeke, 2009; Williams & Collins, 2001). This is important because previous scholars have noted that populations experiencing greater marginalization are more likely to experience loneliness (Madani et al., 2022; Taylor & Nguyen, 2020; Taylor et al., 2023).

In contrast, it is also possible that Black and Hispanic/Latinx older adults may experience less loneliness compared to White older adults. Previous research demonstrates that Black older adults are significantly more likely to have fictive kin relationships than White older adults (Taylor, Skipper, Ellis, et al., 2022). Moreover, Black families also have a greater frequency of providing and receiving informal instrumental support in comparison to White families (Taylor, Skipper, Cross, et al., 2022). Hispanic/Latinx older adults may also report less loneliness than White older adults because of their strong social networks. Undergirding these supportive networks may be the cultural value of Familism (or Familismo), which is a Hispanic/Latinx cultural value in which both close and extended family members provide care and social support to each other (Almeida et al., 2009; Caballero, 2011; Taylor et al., 2013). Though this area of research is underdeveloped, a recent study by Gallegos and Segrin (2022) found that Familism was associated with lower loneliness among Latinx older adults.

Another critical factor that may influence loneliness particularly among Hispanic/Latinx older adults is residing in ethnic enclaves. Ethnic enclaves are geographically defined areas where a greater concentration of a particular ethnic group may reside (Garcia-Hallett et al., 2020; Viruell-Fuentes, 2007). Ethnic enclaves are important because they often serve as a “landing pad” for newly arrived migrants as these communities typically have ethnic-specific businesses (grocery stores that sell Indigenous or culturally specific foods) and Spanish-speaking places of faith and worship, for example. Through the available social networks within these ethnic enclaves, a migrant who resides in these ethnic enclaves may be able to access greater social support and social capital for obtaining jobs, enrolling (or reenrolling) in school/higher education, accessing government programs/assistance, and/or obtaining social services (Garcia-Hallett et al., 2020; Viruell-Fuentes, 2007). Hence, these protective facets of living in ethnic enclaves may reduce feelings of loneliness for some of these Hispanic/Latinx older adults.

### Theory of Fundamental Causes: Income and Education as Mediators for Loneliness

According to the Theory of Fundamental Causes (TFC), structural racism and other macrolevel factors contributing to disadvantage (or fundamental causes) affect access to flexible resources, which profoundly influence health (Link & Phelan, 1995; Phelan & Link, 2015). These resources include money, knowledge, power, prestige, and interpersonal resources such as social support, which may mitigate risk of disease and increase access to health protective resources (e.g., medical treatment) through multiple mechanisms. TFC provides a conceptual framework to understand how income and education may mediate the relationship between race/ethnicity and loneliness. That is, how flexible resources (largely driven by socioeconomic status including levels of income and education) affect differences in loneliness by race/ethnicity. We posit that loneliness may be driven by these flexible resources, whereby people of higher socioeconomic backgrounds are able to access and utilize resources that are protective for loneliness, whereas people of lower socioeconomic status are more likely to be lonely due to constrained access to these flexible resources.

Previous research has found that people with lower-income report greater loneliness than higher income adults and older adults (Kung et al., 2021, 2022; Pinquart & Sorensen, 2001; Shiovitz-Ezra & Leitsch, 2010) and some recent studies demonstrate greater formal education attainment is associated with less loneliness (Barjaková et al., 2023; Dahlberg et al., 2022). Older adults with higher income and greater education may be less lonely due to their social networks, a flexible resource. Older adults with higher socioeconomic status tend to have a greater diversity in the types of relationships within their social networks, they also maintain larger social networks, are more likely to participate in social and group activities, and are less likely to be socially isolated from their network members than those with lower socioeconomic status (Cornwell et al., 2008; Taylor et al., 2023). This is important because older adults who are socially integrated are also less likely to be lonely (Shiovitz-Ezra & Leitsch, 2010; Taylor, 2020). Furthermore, more education is frequently associated with greater earning potential and income over time. Previous research by the Department of Education and other researchers have found that higher levels of educational attainment are correlated with a greater likelihood to be employed and higher median earnings (Boser, 2020; National Center for Education Statistics, 2024; Valletta, 2015; Wolla & Sullivan, 2017).

The compounding nature of education and income may manifest over time whereby greater education may generate more income and wealth and in turn, influence health and well-being (Mirowsky & Ross, 2005; Ross & Wu, 1996). Hence, greater educational attainment (at younger ages) may affect older adults’ greater integration into their social networks and mitigate feelings of loneliness later in life. However, these factors are also disproportionally distributed among older adults, with racially minoritized populations often reporting lower income and fewer years of education than White populations resultant of structural racism (Chatters et al., 2021; Williams & Collins, 2001). Further, Black and Hispanic/Latinx populations experience greater stressors and exposures to stressors including racial discrimination, financial stressors, relationship stressors, and access to health care (Sternthal et al., 2011).

In summation, structural racism contributes to greater health disparities, higher morbidity, lower educational attainment and income, and constrained social networks due to disproportionate rates of incarceration and death among Black and Hispanic/Latinx populations (Bailey et al., 2017; Williams & Collins, 2001). Given these findings, racial and ethnic differences in loneliness may be attributable to these flexible resources that are driven by socioeconomic status (income and education). Moreover, several reviews/studies examining risk factors of loneliness (Barjaková et al., 2023; Hawkley et al., 2008) have also concluded that race/ethnicity and socioeconomic status may be more distal factors which affect loneliness among older adults. We specifically examine the mechanisms that may provide further clarity regarding how loneliness is driven by inequities determined by race/ethnicity and socioeconomic status.

### Current Study

Informed by the TFC, the purpose of the current study is to determine the extent to which income and education may explain racial/ethnic differences in loneliness. To the authors’ knowledge, this study is the first to examine whether socioeconomic factors explain racial/ethnic differences in loneliness among older adults. The strong association between race/ethnicity and socioeconomic status warrants a more granular understanding of how race/ethnicity are linked to loneliness via the pathway of socioeconomic factors. We hypothesize that both income and education, examined together and separately, will mediate racial/ethnic differences in loneliness among older adults.

## Research Design and Methods

### Sample

Data for the current study come from the Health and Retirement Study (HRS), a nationally representative longitudinal panel study of adults aged 50 and older living in the United States. The HRS selects its respondents through a multistage national probability sampling design that features an oversampling for Black and Hispanic older adults, and those residing in Florida. The HRS surveys respondents once every 2 years. Please see Fisher and Ryan (2017) for more information on the HRS and its sampling frame.

In 2006, the HRS initiated enhanced face-to-face interviews following the core interview, which included a self-administered psychosocial and lifestyle questionnaire, also referred to as the leave-behind questionnaire (HRS LBQ). A variety of questions are asked in the HRS LBQ, including social networks and social support, loneliness, personality, and self-rated beliefs, making these data suitable for the study objectives. In 2006, a random half of the HRS core sample was selected for the HRS LBQ. In 2008, the other random half sample was selected to participate. These random half samples participate in the HRS LBQ once every 4 years; therefore, the 2006 participants were interviewed again in 2010, 2014, and 2018, whereas the 2008 participants were interviewed again in 2012 and 2016 (please refer to Smith and colleagues 2017 for further information). For the final analytic sample (*N* = 12,606), we included respondents from the HRS LBQ 2014 or 2016 wave and have data on their race and Hispanic ethnicity. Respondents were excluded if they identified as “Other” and did not identify as Hispanic (*n* = 468), or if they had missing information regarding their race/ethnicity (*n* = 19).

We chose to do a cross-sectional study because we wanted to first establish whether a mediational relationship exists between race/ethnicity, income and education, and loneliness. Secondly, we chose these waves because 2016 is the most recent HRS wave where the sample was replenished prior to the COVID-19 pandemic.

### Variables

Loneliness was measured by the UCLA 3-item loneliness scale (Hughes et al., 2004). These three items include: how often do you lack companionship?; how often do you feel left out?; and how often do you feel isolated? Response options were (1) often, (2) some of the time, and (3) hardly ever or never. Scores from these three items were reverse coded, averaged together, and ranged from 1 to 3, with higher scores indicating a higher level of loneliness. The Cronbach’s alpha coefficient for the UCLA 3-item loneliness scale was 0.81 for the entire sample, 0.81 for White older adults, 0.79 for Black older adults, and 0.80 for Hispanic older adults, indicating good reliability.

Race/ethnicity was operationalized as a combination of two separate variables in the HRS. First, respondents identified their race as White, Black, or Other. Second, respondents identified as having Hispanic ethnicity or not. These two variables were combined, and respondents were identified as non-Hispanic White, non-Hispanic Black, or Hispanic.

Income and education were the key mediators of this study. Income was operationalized as a continuous measure that was log-transformed to reduce skewness. Education was also operationalized continuously by years of education.

Covariates for the current study included gender, age, immigration status, employment status, marital status, living arrangements, country region, and urbanicity. Gender was operationalized into male or female. Age was categorized as 64 and younger, 65 to 74, and 75 and older. Immigration status was measured dichotomously; respondents were either born in the United States or not born in the United States. Employment status was measured as a dichotomous variable, either currently working or not working. Marital status was also a dichotomous variable, either married or unmarried (divorced, separated, widowed, or never married). Living arrangements were measured as living by themselves or living with others. Country regions (South, Northeast, Midwest, and West) was also controlled in the models. Urbanicity of the environment was measured with a single item, urban, suburban, or rural areas. We also controlled for the HRS LBQ waves (2014, 2016).

Sociodemographic measures including gender, age, employment status, living arrangements, marital status, immigration status, country region, and urbanicity were included as covariables due to being associated with key variables in the study. Previous research has found these factors are significantly associated with loneliness among older adults (Barjaková et al., 2023; Buecker et al., 2021; Fang et al., 2021; Fokkema et al., 2012; Hawkley et al., 2022; Henning-Smith et al., 2019; Lim et al., 2020; Luhmann et al., 2023; Pinquart & Sorensen, 2001; Taylor, 2020; Tsuchiya et al., 2023; Wu & Penning, 2015). Furthermore, previous research has also found immigration status (being born in the United States), country region, and urbanicity are often related to race/ethnicity (Krogstad et al., 2022; Moslimani et al., 2024).

### Analysis

All analyses in the current study utilized the HRS-provided complex survey sampling weights and design factors (i.e., stratum and cluster). Accounting for the complex multistage sampling design of the HRS allowed us to generate nationally representative estimates.

To examine if income and education mediated racial/ethnic differences in loneliness, we utilized the Karlson–Holm–Breen (KHB) method (Kohler et al., 2011). Although the KHB method was originally developed for nonlinear models, such a method is also applicable to linear nested models (Kohler et al., 2011). Additionally, the KHB method can decompose the total effect of racial differences in loneliness including both an indirect mediated effect (through income and education) and a direct unmediated effect, illustrating how much of the racial/ethnic differences in loneliness are attributed to income and education.

Of the total 12,606 respondents in our data set, there were 1,186 respondents (about 9% of the sample) with missing data. Variables with missing data included the region variable (783 missing, 6% of the sample), the loneliness variable (209 missing, 1.6% of the sample), the urbanicity variable (191 missing, 1.5% of the sample), and education (49 missing, 0.4% of the sample). To address the missing values in the data, we conducted multiple imputation with chained equations (MICE). MICE is a flexible and robust imputation method that allows for imputation of different variable distributions, including nominal variables, ordinal variables, interval/ratio variables, and count variables (Berglund & Heeringa, 2014). We imputed 20 different data sets in total using MICE. Regression models were estimated separately across each of the 20 imputed data sets. Parameter estimates and standard errors were then combined from each of the 20 data sets to determine statistical significance. Data were analyzed using Stata 18.

## Results

### Descriptive and Bivariate Analyses by Race/Ethnicity

Table 1 shows the univariate and bivariate statistics by race/ethnicity with key study variables. Most respondents identified as White (80%), with approximately 10% identifying as Black and 10% identifying as Hispanic. Most respondents identified as women and were middle-aged (ages 50–64). The average income in the sample was $88,655. Additionally, the average years of education in the sample was 13 years. Most respondents were not employed (53%), married (60%), lived with other people (76%), resided in the South (36%), and in urban areas (50%). A greater percentage of respondents were in the 2016 HRS LBQ wave (55%) compared to the 2014 HRS LBQ wave. The average loneliness score was 1.49.

**Table 1.** Univariate Statistics and Bivariate Statistics for Key Study Variables (*N* = 12,606)

Variables	Total	White	Black	Hispanic	*p* Value
Race/Ethnicity, % (*n*)					
White	79.32% (8,742)				
Black	10.72% (2,280)				
Hispanic	9.96% (1,584)				
Gender, % (*n*)					.002
Male	46.14% (5,113)	46.41% (3,650)	41.76% (799)	48.67% (664)	
Female	53.86% (7,493)	53.59% (5,092)	58.24% (1,481)	51.33% (920)	
Age, % (*n*)					<.001
50–64	51.27% (5,164)	48.94% (2,983)	58.62% (1,270)	61.96% (911)	
65–79	36.85% (5,302)	38.20% (3,955)	33.15% (804)	30.09% (543)	
80 and above	11.88% (2,140)	12.86% (1,804)	8.23% (206)	7.96% (130)	
Employment status, % (*n*)					.005
Not working	54.36% (8,048)	53.63% (5,702)	60.52% (1,433)	53.58% (913)	
Working	45.64% (4,558)	46.37% (3,040)	39.48% (847)	46.42% (671)	
Marital status, % (*n*)					<.001
Married	60.25% (7,184)	63.66% (5,354)	37.66% (870)	57.42% (960)	
Unmarried	39.75% (5,422)	36.34% (3,388)	62.34% (1,410)	42.58% (624)	
Born in the United States, % (*n*)					<.001
No	8.64% (1,369)	3.44% (332)	5.68% (140)	53.21% (897)	
Yes	91.36% (11,237)	96.56% (8,410)	94.32% (2,140)	46.79% (687)	
Living arrangements, % (*n*)					<.001
Living with others	76.29% (9,523)	76.59% (6,573)	67.74% (1,587)	83.14% (1,363)	
Living alone	23.71% (3,083)	23.41% (2,169)	32.26% (693)	16.86% (221)	
Country region, % (*n*)					<.001
Northeast	16.60% (1,748)	17.29% (1,263)	15.50% (325)	11.59% (160)	
Midwest	26.17% (2,861)	29.91% (2,439)	16.56% (374)	3.62% (48)	
South	36.82% (4,870)	33.05% (3,034)	59.73% (1,225)	44.18% (611)	
West	20.41% (2,344)	19.74% (1,604)	8.21% (152)	40.60% (588)	
Urbanicity, % (*n*)					.012
Urban	49.50% (6,376)	47.04% (3,965)	62.69% (1,478)	55.03% (933)	
Suburban	23.20% (2,818)	22.57% (1,941)	19.70% (438)	32.05% (439)	
Rural	27.30% (3,221)	30.39% (2,718)	17.61% (329)	12.92% (174)	
HRS LBQ Wave, % (*n*)					.226
2014	44.79% (6,761)	45.40% (4,815)	43.34% (1,159)	41.47% (787)	
2016	55.21% (5,845)	54.60% (3,927)	56.66% (1,121)	58.53% (797)	
Household income, *Mean (SD)*	88,655.04 (135,638.60)	99,097.56 (136,076.60)	46,503.63 (69,955.80)	50,875.83 (104,750)	<.001
Education, *Mean (SD)*	13.30 (2.96)	13.75 (2.32)	12.62 (3.54)	10.22 (4.99)	<.001
Loneliness, *Mean (SD)*	1.49 (0.55)	1.48 (0.51)	1.57 (0.73)	1.45 (0.61)	<.001

*Notes*: HRS LBQ = Health and Retirement Study Leave-Behind Questionnaire; *SD* = standard deviation. Chi square tests were used to assess racial/ethnic differences for all categorical variables, including gender, age, employment status, marital status, born in the United States, living arrangements, country region, urbanicity, and HRS LBQ Wave. One-way ANOVA tests were used to assess racial/ethnic differences for all continuous variables, including household income, education, and loneliness.

There were significant differences across key study variables by race/ethnicity. White older adults reported higher incomes and greater years of education compared to Black and Hispanicolder adults. Additionally, White older adults were more likely to be older, not employed, and married. Hispanicolder adults were more likely to live with others than Black and White older adults.

### Bivariate Correlations Analyses

Table 2 presents the results of bivariate correlations for the key variables and covariates in our study. We find that household income is significantly correlated with all covariates except for HRS LBQ Wave. Additionally, years of education are also correlated with all covariates except for living arrangements and HRS LBQ Wave.

**Table 2.** Bivariate Correlations

Variables	1	2	3	4	5	6	7
Household income	—						
Education	0.37^***^	—					
Loneliness	−0.15^***^	−0.06^***^	—				
Age	−0.10^***^	−0.11^***^	−0.05^***^	—			
Employment status	0.27^***^	0.20^***^	−0.05^***^	−0.47^***^	—		
Marital status	−0.34^***^	−0.11^***^	0.22^***^	0.12^***^	−0.12^***^	—	
Living arrangements	−0.23^***^	−0.02	0.18^***^	0.16^***^	−0.12^***^	0.67^***^	—
HRS LBQ wave	0.01	0.02	0.01	−0.06^***^	0.06^***^	0.02	0.00

*Notes*: HRS LBQ = Health and Retirement Study Leave-Behind Questionnaire. Pearson’s *R* correlations were used to assess all bivariate correlations.

^***^ *p *< .001.

### Unadjusted and Adjusted Multivariable Models

Table 3 presents the results of multivariable models for loneliness regressed by race/ethnicity. For the unadjusted models, Black older adults had significantly higher levels of loneliness compared to White and Hispanic older adults. White and Hispanic older adults had comparable scores of loneliness. After adjusting for all covariates, Hispanic older adults had significantly lower levels of loneliness compared to White and Black older adults, with no significant difference in loneliness between White and Black older adults.

**Table 3.** Race/Ethnicity and Loneliness Differences Among Older Adults: Unadjusted and Fully Adjusted Models

Race/Ethnicity	Margins	White reference	Black reference	Hispanic reference
Unadjusted model				
White	1.48^a^	—	−0.09 (−0.12, −0.05)^***^	0.03 (−0.01, 0.07)
Black	1.57^a^	0.09 (0.05, 0.12)^***^	—	0.11 (0.07, 0.16)^***^
Hispanic	1.46^a^	−0.03 (−0.07, 0.01)	−0.11 (−0.16, −0.07)^***^	—
Fully adjusted model				
White	1.50^b^	—	0.01 (−0.02, 0.05)	0.08 (0.03, 0.13)^**^
Black	1.49^b^	−0.01 (−0.05, 0.02)	—	0.07 (0.02, 0.11)^**^
Hispanic	1.42^b^	−0.08 (−0.13, −0.03)^**^	−0.07 (−0.11, −0.02)^**^	—

*Notes*: Unstandardized regression coefficients (95% confidence intervals) are presented. Fully adjusted models control for gender, immigration status, age, income, education, employment status, marital status, living arrangements, country region, and urbanicity.

^a^Unadjusted margins are reported.

^b^Adjusted margins are reported.

^*^ *p *< .05;

^**^ *p *< .01;

^***^ *p *< .001.

### Mediation Analysis

The results of the KHB mediation analysis are shown in Table 4. In comparing differences in loneliness between White and Black older adults, full mediation was observed, with significant indirect effects of income and education. Said another way, income and education fully mediated differences in loneliness between Black and White older adults. A partial mediation was observed in the comparisons between White and Hispanic older adults and Black and Hispanic older adults, where both significant direct and indirect effects were observed. Additionally, approximately 120% of the difference in loneliness between Black and White older adults is due to income and education, whereas 160% of the difference in loneliness between White and Hispanic older adults was accounted for by income and education. Forty-seven percent of the difference in loneliness between Black and Hispanic older adults is due to income and education.

**Table 4.** Income and Education Mediation Models Explaining Racial/Ethnic Differences in Loneliness Among Older Adults

Race/ethnicity	Income and education mediation model		Income mediation model		Education mediation model	
	Decomposed effects of income and education	Confounding percentage	Decomposed effects of income	Confounding percentage	Decomposed effects of education	Confounding percentage
*With White as comparison*						
Black		119.70 %		133.64%		−429.58%
Total effect	0.02 (0.02)		0.01 (0.02)		−0.00 (0.02)	
Direct unmediated effect	−0.00 (0.02)		−0.00 (0.02)		−0.00 (0.02)	
Indirect mediated effect	0.02 (0.01)^***^		0.02 (0.00) ^**^		0.00 (0.00)	
Hispanic		−160.10%		−38.44%		−18.24%
Total effect	−0.03 (0.02)		−0.06 (0.02)^*^		−0.06 (0.02)^**^	
Direct unmediated effect	−0.08 (0.02)^**^		−0.08 (0.02)^**^		−0.08 (0.02)^**^	
Indirect mediated effect	0.05 (0.01)^***^		0.02 (0.01)^***^		0.01 (0.01)	
*With Black as comparison*						
White		119.70%		133.64%		−429.58%
Total effect	−0.02 (0.02)		−0.01 (0.02)		0.00 (0.02)	
Direct unmediated effect	0.00 (0.02)		0.00 (0.02)		0.00 (0.02)	
Indirect mediated effect	−0.02 (0.01)^***^		−0.02 (0.00)^**^		−0.00 (0.00)	
Hispanic		−46.75%		−8.32%		−13.45%
Total effect	−0.05 (0.02)^*^		−0.07 (0.03)^**^		−0.06 (0.02)^**^	
Direct unmediated effect	−0.07 (0.03)^**^		−0.07 (0.03)^**^		−0.07 (0.03)^**^	
Indirect mediated effect	0.02 (0.01)^***^		0.01 (0.00)		0.01 (0.00)	

*Note*s: Unstandardized coefficients (standard errors) are presented. Mediation models adjusted for all covariables, including gender, immigration status, age, income, education, employment status, marital status, living arrangements, country region, and urbanicity.

^*^ *p *< .05.

^**^ *p *< .01.

^***^ *p *< .001.

Confounding percentages greater than 100% signifies that the indirect mediated effect is larger than the combined total effect (where the total effect is the combined indirect and direct effect). These results were observed in the current study because the direct unmediated effect and the indirect mediated effect were in opposite directions (i.e., the direct effect is positive and the indirect effect is negative, and vice versa). When the direct effect and indirect effect are combined, this contributes to a smaller total effect and hence, may result in confounding percentages greater than 100%. Other scholars have provided further clarifications for these types of mediational analyses (see VanderWeele & Vansteelandt, 2014).

We conducted additional mediation models examining income and education separately. Racial/ethnic differences in loneliness among White and Black older adults were fully mediated by income, with income partially mediating the relationships between White and Hispanic older adults. Income did not mediate racial/ethnic differences in loneliness between Black and Hispanic older adults. In contrast, education did not account for any mediation effects (partial or full) in loneliness across all comparisons of racial/ethnic groups. Please see Table 4 for more details.

In supplemental analyses, we also tested if wealth also mediated racial/ethnic differences in loneliness among older adults. When solely examining wealth, we find similar results to the main results with income with wealth fully mediating racial differences in loneliness between White and Black older adults. Moreover, we found wealth partially mediates racial/ethnic differences in loneliness between White and Hispanic older adults and Black and Hispanic older adults. Please see Supplementary Table 1 for these specific results.

## Discussion and Implications

Our study is the first to examine if and whether income and education mediate differences in loneliness by race/ethnicity among older adults. Our study found evidence of both total and partial mediation effects among White, Black, and Hispanic older adults.

### Full Mediation Effects for Explaining Race/Ethnic Differences in Loneliness Among Black and White Older Adults

In comparing White and Black older adults, we found a total mediation effect. Said another way, the significant differences in loneliness between White and Black older adults are attributable to income and education. This further illustrates the importance of controlling for income and education in multivariable statistical models when examining racial/ethnic differences in loneliness. In further analyses, we found income was the key mediator that explained these differences between White and Black older adults. Education did not mediate the relationship between race/ethnicity and loneliness among White and Black older adults.

Our findings may be explained by the fact that older adults with limited income may be more distressed (Tsuchiya et al., 2020) due to experiencing multiple financial stressors and in turn, feel lonelier than older adults with greater income. Having greater household income reduces the overall economic and financial burden on a family. Indeed, research has found families with greater household income experience less loneliness and especially among older adults (de Jong Gierveld & Tesch-Römer, 2012). This is because older adults living in households with greater incomes may be able to actively choose who they want to interact with or rely on for their social and emotional support needs, resulting in more positive social interactions among family members. In contrast, older adults from lower-income households may rely more on family members and friends for informal support, which may also contribute to difficulties in potentially distancing oneself from negative or stressful relationships as they may also be unable to focus on investing in positive relationships. These challenging relationships may lead to significantly greater conflict and negative interactions, and in turn, more loneliness (de Jong Gierveld & Tesch-Römer, 2012).

As well, less income or money may limit the number of social activities and events that an older adult can participate in, whereas having access to more money may contribute to more options for social integration and resources for leisurely activities (e.g., going out to dinner and participating in clubs). These contextual factors may ultimately lead to less loneliness (Barjaková et al., 2023; Chatters Taylor, 2019; Pinquart & Sorensen, 2001). Previous research has also found significant income-loneliness association regardless of work status, social engagement, number of friends, and frequency of contact (Hawkley et al., 2022; Luhmann & Hawkley, 2016; Pinquart & Sorensen, 2001) suggesting a broader social context that contributes to both lower income and increased loneliness.

When comparing White and Black older adults in the current sample, White older adults were generally less lonely and reported greater income in comparison to Black older adults. These findings support pathways outlined in the TFC, whereby having greater access to flexible resources (especially income) can facilitate and reduce loneliness among older adults through multiple mechanisms. In other words, our findings demonstrate that these racial/ethnic differences are manifestations of racism that have cascading impacts for loneliness via money, a flexible resource, for Black older adults. The historical inequitable distribution of power/resources among Black communities translates into constrained access to flexible resources leading to greater risks for loneliness.

### Partial Mediation Effects in Explaining Race/Ethnic Differences in Loneliness for Older Adults

When comparing both White and Hispanic older adults and Black and Hispanic older adults, we found a partial mediation effect of both income and education, in which part of the racial/ethnic differences in loneliness were attributable to these socioeconomic factors. This may be explained by the fact that there are likely additional factors, such as cultural differences and/or expectations, differences in environmental contexts, or variability in psychosocial resources, which may explain these differences in loneliness. The adjusted models demonstrate that Hispanicolder adults report the lowest levels of loneliness than other groups in this sample. Hispanic/Latinx older adults may have stronger bonds and connections with greater social support because of familismo and/or through living in primarily Hispanic/Latinx ethnic enclaves (Almeida et al., 2009; Caballero, 2011; Gallegos & Segrin, 2022; Garcia-Hallett et al., 2020; Viruell-Fuentes, 2007; Taylor et al., 2013). Although Hispanic/Latinx older adults may report lower income than their White counterparts, as informed by the TFC, strong social connections and community ties may potentially mitigate some of the negative consequences of limited income for loneliness among Hispanic/Latinx older adults. As an initial investigation in understanding differences in loneliness by race/ethnicity, we invite future research to examine additional factors that may mediate these relationships.

### Education Mediation Effects

In examining income and education separately in the mediation models, education did not explain differences in loneliness by race/ethnicity in any of the models. In considering previous literature, which has illustrated differences in educational attainment by race/ethnicity, we anticipated that education would account for some of the variability in loneliness by race/ethnicity. Our findings can be explained through several potential reasons. First, education has been shown to have mixed/inconsistent findings with loneliness (Barjaková et al., 2023). Previous studies have found that as education may have an inverse/negative relationship with loneliness in bivariate relationships, but this association may no longer be significant or may become positive when other relevant sociodemographic covariates (i.e., income, employment status, gender, and age) are included in multivariable analyses (Dahlberg et al., 2018; Luhmann & Hawkley, 2016; Nicolaisen & Thorsen, 2014).

Second, educational attainment may have a different association with loneliness depending on one’s race/ethnicity. For example, studies among Black older adults have found that greater education is related to greater isolation from adult children and other family members, greater likelihood of living alone, and less participation in neighborhood social groups (Taylor et al., 2019, 2023). Having limited contact with family members (potentially because of the quality of these social relationships) and living alone are noted to be among some of the strongest factors that influence loneliness (Barjaková et al., 2023; Hawkley et al., 2008). Hence, these aforementioned factors may place Black older adults at greater risk of being lonely. Third, it is important to consider the life course perspective in situating our findings (Elder et al., 2003; Gee et al., 2012). In considering the concept of sensitive periods, whereby stressors or critical life events have a salient impact on health when they occur during specific developmental stages, education may not be as salient for loneliness among older adults due to their current stage in life. Previous research has found that racism as a byproduct of residential and school segregation affects the development of social connections and relations and in turn employment opportunities (Wagmiller, 2007). One study found that youth who graduated from high schools that were primarily composed of racialized youth were more likely to also work in jobs that employed primarily racially minoritized groups even after accounting for region, school, and segregation (Stearns, 2010). Hence, educational attainment and where they enroll may shape Black older adults’ social networks earlier in life, which has profound consequences for their social connections and relationships in older age, and ultimately, contributes to loneliness.

Given the complexity of the role of education for loneliness, it will be important for future empirical research (including qualitative studies) to contextualize the influence of education on loneliness to specific communities and populations. Additionally, more research is needed to understand how income/wealth, social network resources and contact, living arrangements, chronic and temporary stressors, and relationship quality may also influence the relationship between education and loneliness across diverse racial/ethnic groups (Hawkley et al., 2008) and ultimately, how mechanisms of structural racism affect these associations.

### Limitations

Several limitations should be considered in this study. First, the relationships were not causal due to the cross-sectional design adopted in this study. Second, this study only examines one aspect of loneliness. Research by De Jong Gierveld and Van Tilburg (2006) has shown that there are two different types of loneliness: emotional and social, whereas Cacioppo and colleagues (2015) hypothesized that there are three types of loneliness: intimate loneliness, relational loneliness, and collective loneliness. Given that the UCLA loneliness scale is constructed as a unidimensional measure (Russell, 1996), further differentiations into varied types of loneliness would not be feasible. Said another way, socioeconomic factors may or may not mediate the relationship between race/ethnicity and other forms of loneliness (i.e., intimate loneliness). Third, the current study did not assess the chronicity of loneliness, or divide participants into those who are temporarily/situationally lonely versus those who are chronically lonely. This is important because (1) there could be different rates of chronic loneliness based on race/ethnicity, and (2) socioeconomic factors may not explain these racial/ethnic differences in loneliness.

Fourth, as outlined by VanderWeele and Vansteelandt (2014), the reason why there are confounding percentages greater than 100% may be because there are missing mediators other than income and education in our analysis. This may include other important downstream and proximate factors that may have a stronger and more direct relationship with loneliness, including frequency of social network contact, social support, negative interactions, frequency of engagement and social participation, and the quality of social relationships (Barjaková et al., 2023; Hawkley et al., 2008). We encourage future research studies to consider these important mediators as they may further explain racial/ethnic differences in loneliness among older adults.

Fifth, not all racial and ethnic identities were included in the current study. For example, Asian and Native American older adults are subsumed under the category “Other” and were not included in the current study. Sixth, this study does not include the racial/ethnic composition of neighborhoods where respondents resided. This may also influence loneliness, as older adults who reside in neighborhoods with similar racial, ethnic, and/or cultural backgrounds may feel less lonely. Lastly, our study does not examine relative deprivation of neighborhoods as it relates to racial differences in loneliness among older adults. Previous research has found neighborhood deprivation is another important socioeconomic factor to examine (Kearns et al., 2015a, 2015b; Scharf & de Jong Gierveld, 2008), and this could be especially important given different experiences of living in racially segregated neighborhoods, different community norms, and available local neighborhood amenities.

### Implications

Income is a flexible resource that influences multiple domains; hence, the relationship between income and loneliness is often considered a distal factor influencing loneliness. Loneliness is likely to be mediated/moderated by other factors including individual health/well-being, accessible public or personal transportation, and the frequency of contact and exchange of social support among network members (Barjaková et al., 2023; Hawkley et al., 2008; Pinquart & Sorensen, 2001; World Health Organization, 2021). Barriers to socialization for racialized and/or low-income older adults may be addressed through interventions that improve connection, including peer-to-peer, volunteer, and community navigator interventions.

Previous studies demonstrate peer-to-peer outreach and volunteer interventions are effective at decreasing loneliness among racially/ethnically diverse and low-income older adults (Cao et al., 2023; Fuller et al., 2022; Kotwal et al., 2021). Key elements of these programs include nurturing opportunities for socialization and developing trust and social capital among intervention workers/volunteers and participants (who are often matched based on sociodemographic background and similar interests). Developing community navigator programs and partnering with local community-based organizations, which serve racialized and low-income older adults (including churches and senior centers) may also be effective in mitigating loneliness among the most marginalized older adults, therefore decreasing racial/ethnic inequities in loneliness (Smith et al., 2023; Stefanidou et al., 2023).

## Supplementary Material

igae068_suppl_Supplementary_Table

## Data Availability

Data used for this study come from the Health and Retirement Study, and these data used are publicly available. This data can be accessed from https://hrs.isr.umich.edu/. The analytic methods, used for replication purposes, are available to other researchers upon request to the first author. None of the studies included in this manuscript, including the current study, were preregistered.
