# Supplementary material for: Racial/Ethnic Differences in Loneliness Among Older Adults: The Role of Income and Education as Mediators
Source: Innov Aging. 2024 Jul 19;8(8):igae068. doi: 10.1093/geroni/igae068 (PMC11319872; doi:10.1093/geroni/igae068)
Supplement: igae068_suppl_Supplementary_Table [file igae068_suppl_supplementary_table.docx]

| **Supplemental Table 1. Income, Education and Wealth Mediation Models Explaining Racial/Ethnic Differences in Loneliness among Older Adults** | | | | |
| --- | --- | --- | --- | --- |
|  | **Income, Education and Wealth Mediation Model** | | **Wealth Only Mediation Model** | |
| **Race/Ethnicity (with White as comparison)** | **Decomposed Effects of Income, Education, and Wealth** | **Confounding Percentage %** | **Decomposed Effects of Wealth Only** | **Confounding Percentage** |
| **Black** |  | 221.87 % |  | -518.64% |
| Total Effect | 0.02 (0.02) |  | -0.00 (0.02) |  |
| Direct Unmediated Effect | -0.02 (0.02) |  | -0.02 (0.02) |  |
| Indirect Mediated Effect | 0.05 (0.01)*** |  | 0.02 (0.01)*** |  |
| **Hispanic** |  | 198.59% |  | -14.80% |
| Total Effect | -0.03 (0.02) |  | -0.08 (0.02** |  |
| Direct Unmediated Effect | -0.09 (0.02)*** |  | -0.09 (0.02)*** |  |
| Indirect Mediated Effect | 0.06 (0.01)*** |  | 0.01 (0.00)** |  |
|  | **Income, Education and Wealth Mediation Model** | | **Wealth Only Mediation Model** | |
| **Race/Ethnicity (with Black as comparison)** | **Decomposed Effects of Income, Education, and Wealth** | **Confounding Percentage %** | **Decomposed Effects of Wealth Only** | **Confounding Percentage** |
| **White** |  | 221.87% |  | -518.64% |
| Total Effect | -0.02 (0.02) |  | 0.00 (0.02) |  |
| Direct Unmediated Effect | 0.02 (0.02) |  | 0.02 (0.02) |  |
| Indirect Mediated Effect | -0.05 (0.01)*** |  | -0.02 (0.01)*** |  |
| **Hispanic** |  | -28.26% |  | 12.60% |
| Total Effect | -0.05 (0.02)* |  | -0.07 (0.02)** |  |
| Direct Unmediated Effect | -0.06 (0.02)* |  | -0.06 (0.02)* |  |
| Indirect Mediated Effect | 0.01 (0.01) |  | -0.01 (0.00)* |  |
| **Note:**. * indicates statistical significance at the 0.05 level, and *** indicates statistical significance at the 0.001 level. | | | | |
